# Advanced modification of drug nanocrystals by using novel fabrication and downstream approaches for tailor-made drug delivery

**DOI:** 10.1080/10717544.2019.1682721

**Published:** 2019-11-18

**Authors:** Tao Liu, Xinxin Yu, Haipeng Yin, Jan P. Möschwitzer

**Affiliations:** a Department of Pharmaceutical Engineering, Qingdao University of Science and Technology, Qingdao, China;; b Department of Internal Medicine, Qingdao orthopaedic Hospital, Qingdao, China;; c Institute of Pharmacy, Department of Pharmaceutics, Biopharmaceutics and NutriCosmetics, Freie Universität Berlin, Berlin, Germany

**Keywords:** Drug nanosuspension, modification of drug nanocrystals, tailor-made drug delivery

## Abstract

Drug nanosuspensions/nanocrystals have been recognized as one useful and successful approach for drug delivery. Drug nanocrystals could be further decorated to possess extended functions (such as controlled release) and designed for special in vivo applications (such as drug tracking), which make best use of the advantages of drug nanocrystals. A lot of novel and advanced size reduction methods have been invented recently for special drug deliveries. In addition, some novel downstream processes have been combined with nanosuspensions, which have highly broadened its application areas (such as targeting) besides traditional routes. A large number of recent research publication regarding as nanocrystals focuses on above mentioned aspects, which have widely attracted attention. This review will focus on the recent development of nanocrystals and give an overview of regarding modification of nanocrystal by some new approaches for tailor-made drug delivery.

## 1. Introduction

Drug nanosuspensions, often referred to as drug nanocrystals, can be considered as one of the most successful pharmaceutical nanotechnology approach. Nanocrystal technology has gone through nearly 30 years of evolutionary improvement since it was firstly invented in 1991 (Muller & Keck, 2012). To date, an increasing number of novel advanced preparation methods and downstream technologies have been developed for tailor-made drug delivery. Consequently, the drug nanocrystal preparation efficiency has been improved and its functional characters have also been highly extended.

Regarding the preparation methods, the top-down (high pressure homogenization and wet bead milling) and bottom-up (solvent antisolvent precipitation) methods are still the most frequently used preparation approaches (Liu et al., 2015; Gigliobianco et al., 2018; Malamatari et al., 2018; Miao et al., 2018). Some novel technologies have been invented recently, which possessed the capability to produce smaller nanoparticle sizes (e.g. <100 nm) (Li et al., 2015), improve the production efficiency (Liu et al., 2016) or achieve pure drug nanoparticles of amorphous active pharmaceutical ingredients (APIs) (Guo et al., 2013). Most of the new invented technologies are still based on the top-down or bottom-up approaches.

Drug nanosuspension is a relatively “simple” system, which is consisted of only API dispersed in aqueous stabilizer solutions (Peltonen & Hirvonen, 2018). Although just a particles size reduction into the nanometer range can by itself lead to a faster dissolution rate or a higher saturation solubility compared to microsized API, this technology normally cannot possess advantages such as targeting compared to other nanocarrier systems. Most recently, a lot of research groups have started to use downstream processes to broaden its capabilities and applications. Drug nanocrystals have been decorated to further possess advanced in vitro and in vivo performances based on the specific requirement of drug delivery.

**Figure 1.**
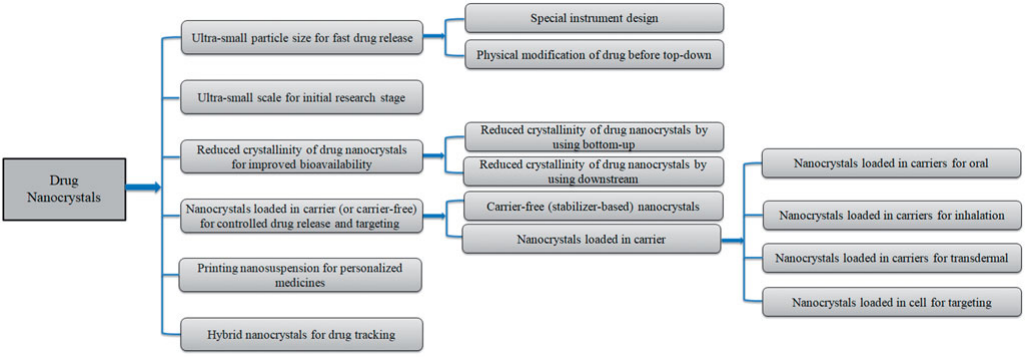
Schematic description of modified nanocrystals by using different preparation and downstream processes for various tailor-made drug deliveries.

This review will mainly focus on the most recent development of drug nanocrystals regarding as novel specific aims of drug delivery or modified in vitro as well as in vivo performances based on some new preparation technologies or downstream processes (Figure 1), as most research publication in recent years about drug nanocrystals are correlated to those aspects (Table 1).

**Table 1.** overview of examples of advanced nanocrystal preparation technologies and downstream processes.

APIs	Particle size reduction methods	Particle size of nanosuspensions(nm)	Downstream processes	Reference
griseofulvin	WBM using very small beads (50 µm)	88		(Li et al., 2015)
4 APIs	WBM of ultra-small scale (5 mg/batch)	200–500		(Liu et al., 2017)
caffeine	WBM using organic media	250		(Zhai et al., 2014)
10-hydroxycamptothecin	Prec + HPH	131		(Pu et al., 2009)
amphotericin B	Prec+HPH	21		(Sinha, 2013)
glibenclamide	FD + HPH	182		(Salazar et al., 2011)
glibenclamide	FD + WBM	199		(Salazar et al., 2012)
glibenclamide	SD + HPH	258		(Salazar et al., 2013)
resveratrol	SD + HPH	192		(Liu et al., 2016)
baicalin	cellulose–sodium carboxymethyl starch used as stabilizer	846		(Xie et al., 2019)
lovastatin	alginate used as stabilizer	370		(Guan et al., 2017)
clarithromycin	Prec + FD + HPH	460		(Morakul et al., 2013)
curcumin	Prec with a multi-inlet vortex mixer	20		(Bi et al., 2017)
furosemide	Büchi nano spray-dryer B-90	1245		(Li et al., 2010)
atovaquone	pH Based Prec	297		(Kathpalia et al., 2019)
indomethacin	Prec using food protein as stabilizer	100–400		(He et al., 2013)
paclitaxel	Prec using copolymer as stabilizer	236		(Cao et al., 2019)
curcumin	Acid-base reaction method	100		(Wang et al., 2017)
amphotericin B	Amorphous prec	135		(Zu et al., 2014)
silymarin	Nanoporous membrane extrusion	83		(Guo et al., 2013)
resveratrol	HPH	559–625	Nanocrystals was loaded in microparticle by SD for inhalation.	(Liu et al., 2019)
cholecalciferol	Prec	302	Nanocrystals was loaded in microneedle for transdermal.	(Vora et al., 2018)
Curcumin	Prec	ca.400-1000	Nanocrystals was loaded in microneedle for transdermal.	(Abdelghany et al., 2019)
amphotericin B	FD + HPH	65	Encapsulated nanocrystals in human erythrocytes to prevent the drug’s toxicity on the phagocytosing cells.	(Staedtke et al., 2010)
dexamethasone	WBM	272	FD was used to dry and reduce the drug crystallinity	(Colombo et al., 2017)
andrographolide	WBM	244	Amorphization was found after FD processes. Improved bioavailability was observed.	(Qiao et al., 2017)
darunavir	WBM	295	Coaxial electrospraying was used to encapsulate nanocrystals within polymer shell.	(Nguyen et al., 2017)
naproxen	WBM	370	Fluidized bed pellet coater achieved the drug release within 5 min.	(Kayaert et al., 2011)
itraconazole	WBM	<200	Fluid bed coating was used to coat nanosuspensions onto beads. The fastest dissolution rate was from small sugar beads size, HPMC VLV as film polymer and lowest layering level.	(Parmentier et al., 2017)
efavirenz	HPH	320	Drug nanosuspension was mixed with polymer using a twin-screw extruder to get nanocrystal solid dispersions.	(Ye et al., 2016)
clotrimazole	Pre	/	hot melt extruder coupled with polymer matrix was used to dry amorphous nanosuspension.	(Gajera et al., 2018)
folic acid	HPH	407	Nanosuspension was used as printing ink and was printed on edible paper carriers. An example of personalized medicine.	(Pardeike et al., 2011)
paclitaxel	anti-solvent method	380	Fluorescent dyes were combined with nanocrystals for in vivo disease imaging.	(Hollis et al., 2014)
quercetin	HPH	753	Drug nanocrystals were loaded into fast dissolving maltodextrins films, which showed faster dissolution rate than the freeze-dried nanocrystals.	(Lai et al., 2015)

WBM: wet bead milling; Prec: precipitation; FD: freeze-drying; SD: spray-drying; HPH: high pressure homogenization.

## 2. Tailor-made drug delivery based on nanocrystals

### 2.1. Ultra-small particle size for fast drug release

Top-down and bottom-up technologies are the two fundamental approaches for nanocrystal production, which normally lead to particle size in the range of 100 nm–1000 nm. Besides standard top-down and bottom-up methods, this review gives a summary of some recent improved or advanced technologies for ultra-small particle size, i.e. <100 nm.

Wet bead milling (WBM) and high pressure homogenization (HPH) are the two main top-down approaches. WBM uses milling machine to rotate the small milling bead/ball either by an agitator blade or by stirring of the whole sample container. For HPH, the high pressure homogenizer can be divided in two main types: piston-gap and jet-stream. Mechanisms of both have been detailed discussed in many related review papers (Van Eerdenbrugh et al., 2008; Liu et al., 2015; Fontana et al., 2018; Jermain et al., 2018; Couillaud et al., 2019; Pardhi et al., 2019). Normally, no organic solvent is required during the production process compared to the bottom-up processes. However, the substitution of water could be acceptable for some specific purposes (Zhai et al., 2014).

By far, the NanoCrystal^®^ technology based on WBM approach is the most widely applied technology in the pharmaceutical industry (Goel et al., 2019). Top-down technologies usually apply physical force, such as shear force and impact force to diminish the drug particle size from micro-size to nano-size. Specific instrument such as high pressure homogenizer with sufficient pressure (normally more than 1300 bar) is essential to achieve a homogenous particle size in the obtained nanosuspension. Therefore, the design of applied top-down machine is critical for the efficiency of nanocrystal production. In generally, WBM could lead to smaller particle size compared to HPH processes according to the literature (Moschwitzer, 2013). Different instruments could reach various minimal achievable particle sizes for the same drug due to the different instrument design.

The nanocrystal preparation method could also affect the downstream formulation process, therefore, a detailed selection of the approach for production is essential. For instance, meloxicam nanosuspensions were achieved by using three different method, i.e. WBM, HPH and a combination of freeze-drying coupled with HPH. The WBM led to the smallest particle size of 88 nm as well the highest final drug release, however, tablets produced applying nanocrystals obtained from HPH and the two step approach could achieve faster dissolution rates within the first 20 min than WBM (Liu et al., 2018).

#### 2.1.1. Special instrument design

The efficiency of particle size reduction during top-down process is highly dependent on the applied method or instrument. For the same API, different instruments can lead to various particle sizes of nanosuspensions even if it was processed under the same processes parameters, such as homogenization pressure and time, because of the different internal designs. Many studies now are attempting to improve the performance of top-down technologies based on the different principles of the top-down approach, which has widely attracted attention. In order to improve the efficiency of top-down processes or achieve special properties of nanosuspensions, many advanced and optimized technologies focus on the instrument design. Although the commercial instruments in general use the main principles mentioned in the top-down section, which means the core part of those instrument would be similar if the same mechanism was applied, some novel designed processes have been also invented and investigated recently. For example, the milling size of applied beads could affect the final particle size and smaller bead size would lead to smaller nanocrystal size. Based on this theory, it was reported that a WBM instrument allowed to use very small beads size of 50 µm (much smaller than commonly used bead size of 0.4 mm), which could significantly improve the milling efficiency, reduce the contamination and lead to sub-100 nm particle size (Li et al., 2015). Material of bead could be also substituted. In a recent study, ice beads were used as milling media, which could dissolve in water after the milling process and therefore contamination from the milling beads could be avoided (Funahashi et al., 2019).

The bottom-up technologies are generally based on mechanism of solvent-antisolvent precipitation. The API is first dissolved in a solvent and subsequently a precipitation is induced by adding the API-containing solvent phase to a non-solvent, which leads to drug nanocrystals (Liu et al., 2016). Process parameters such as solvent/antisolvent ratio, mixing type and rate, solvent as well as stabilizer selection are critical to achieve a homogenous nanosuspension (Sinha et al., 2013). In general, the bottom-up approach required no high energy input compared to the top-down process. However, organic solvent is normally involved as the drug solvent and must be removed before the final formulation process. For bottom-up approach, the way of solvent and antisolvent mixing could affect the nanoparticle size. Most studies used a syringe containing drug dissolved in solvent, which is injected in the antisolvent (Rahim et al., 2019). Ultrasound or magnetic stirrer could also be simultaneously applied in order to get a homogenous size distribution. One improved method was capable of mixing the solvent and antisolvent inside the high pressure homogenizer, which could result in much smaller size compared to normal precipitation operation (Sinha et al., 2013). The precipitation with a multi-inlet vortex mixer is another modified bottom-up process, which allows the API solvent and anti-solvent to enter a special designed mixer in different directions. This method could effectively control the particle growth with the aid of stabilizer and the particle size of obtained nanosuspensions was smaller than 100 nm. For example, a very small particle size (20 nm) of curcumin was achieved by using this technology. This study also confirmed that the 20 nm curcumin nanosuspensions showed comparable biodistribution to curcumin solution in most tissues during animal experiment (Bi et al., 2017).

Besides top-down and bottom-up technologies, some novel approaches such as acid-base reaction based method were also invented. Drug nanosuspensions can be achieved by using the rapid generated CO_2_ bubbles during the acid-base reaction. For example, curcumin, stabilizer and acid were dissolved in organic solvent and then it was dried by using rotary evaporation to obtain an acid-phase. The carbonate solution was finally added in the acid-phase to achieve curcumin nanosuspension. Particle sizes of around 100 nm could be produced by using different stabilizers and 6 h’ stability was also confirmed (Wang et al., 2017). Like top-down approach, this method uses the external energy (from chemical reaction) to reduce the drug particle size, but it doesn’t need any special energy input equipment. Normally, the selected solvent used for bottom-up process is organic solvent and antisolvent is water. However, the change of pH resulting in the solubility alteration could also be applied as a novel bottom-up method (Li et al., 2018). Atovaquone nanosuspension was reported to be prepared by using this concept and it could have a long stability within 6 months (Kathpalia et al., 2019).

#### 2.1.2. Physical modification of drug before top-down

In addition, physical properties of drug could finally determine the smallest particle size obtained, which means there is always a limitation no matter which kind of instrument is used. For example, it is normally difficult for a lot of APIs to get a particle size smaller than 100 nm by using HPH technologies (Moschwitzer, 2013). Therefore, some advanced technologies have been trying to apply modified drug with improved physical properties for top-down process.

Drug physical properties have significant influence on the particle size reduction effectiveness (Liu et al., 2015, 2018) and the consequent dissolution rate. The unmodified APIs from the synthesis employed for the top-down process can be firstly treated by different modification procedures, e.g. precipitation (Sinha et al., 2013; Singh et al., 2018; Homayouni et al., 2019), spray-drying (Salazar et al., 2013; Liu et al., 2016), freeze-drying (Salazar et al., 2012), high speed homogenization (Oktay et al., 2019) or WBM (Bartos et al., 2018). These modification processes combining with subsequent top-down steps are commonly referred to as combination processes. For example, resveratrol and sodium cholate was firstly dissolved in ethanol and processed by spray-drying. The spray-dried resveratrol powder could highly improve the followed HPH efficiency and only 5 homogenization cycles were enough to obtain the smallest particle size of 195 nm. Conversely, the standard HPH using raw resveratrol could only lead to a particle size of 569 nm after 20 cycles (Liu et al., 2016).

The direction to modify API has not been answered by published studies to date. Based on the limited research, the mechanism to obtain drug with improved milliability is still unclear and needs further systematical investigation (Liu et al., 2015).

**Figure 2.**
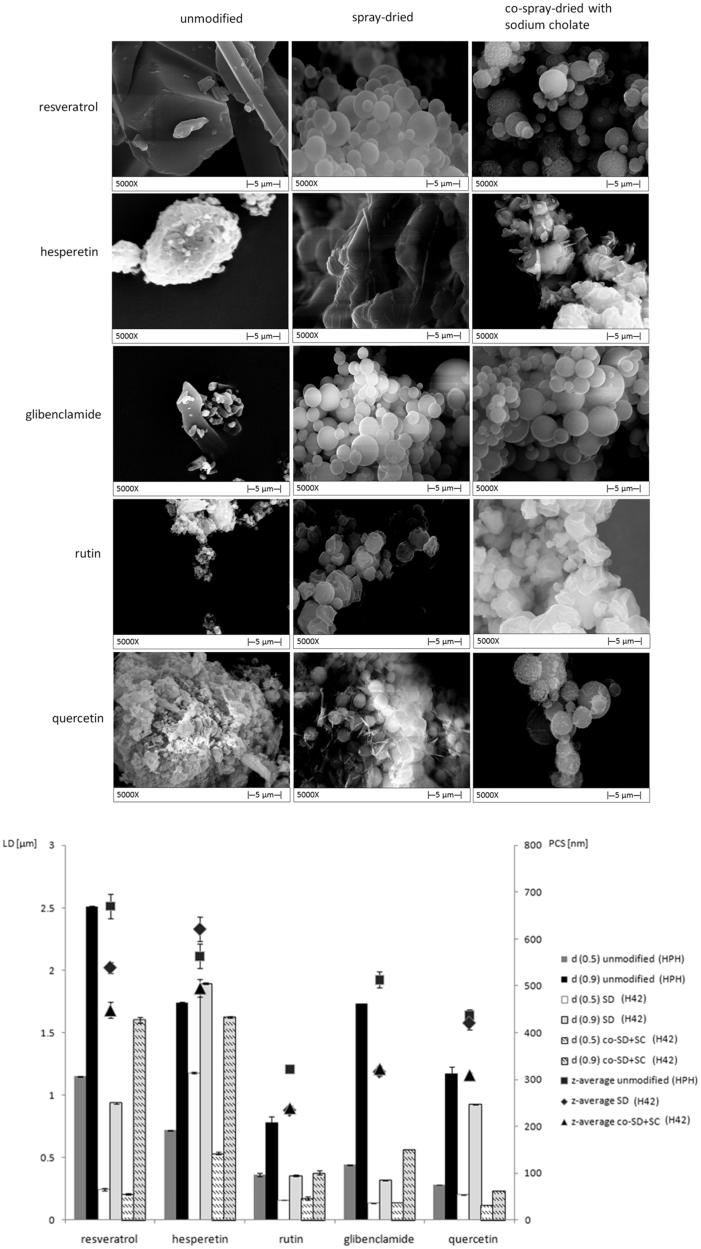
Correlation between particle morphologies and their particle sizes after 20 homogenization cycles at 1500 bar of 5 APIs (LD: laser diffractometry; PCS: photon correlation spectroscopy; SD: spray drying; SC: sodium cholate). Modified after reference (Liu et al., 2018).

Physical properties, such as solid state, particle morphology, particle size, crystallite size and Young’s modulus, have been studied of the correlation about milliability. Table 2 gives a summary of physical properties that may influence the top-down production. For example, some related literature has proved that the modification of drug physical properties such as particle morphology (George & Ghosh, 2013; Liu et al., 2016) might have direct correlation to the particle size reduction efficiency and the minimal achievable particle size. In one recent study, five different kinds of APIs were modified (i.e. spray-dried) under the same experimental conditions. A porous structure or reduced crystallite size observed from the SEM was speculated to have direct correlation with the HPH efficiency, because the only one API, i.e. hesperetin, without any change of particle morphology after direct spray-drying process, was not shown contribution to the followed top-down process (Figure 2) (Liu et al., 2018). In addition, the solid state (Salazar et al., 2011; Liu et al., 2018), polymorphism (Sharma et al., 2011) and Young’s modulus (Cerdeira et al., 2011) of certain APIs were also found possible effect on the top-down processes, among which solid state was attracted a lot of attentions recently.

**Table 2.** possible factors influencing efficiency of particle size reduction.

API property	Reference
particle size/ crystallite size	(Liu et al., 2018)
polymorphism	(Sharma et al., 2011)
particle morphology	(Liu et al., 2016, 2018, 2018)
solid state	(Salazar et al., 2011, 2013; Liu et al., 2018)
Young’s modulus	(Cerdeira et al., 2011)

Amorphous state or reduced crystallinity obtained from such as spray-drying or freeze-drying means the irregular molecular arrangement compared to the crystal state, which could also be used for HPH or WBM processes (Kuroiwa et al., 2018). For crystal API, the breakage points of the powder during top-down processes happened at boundaries of the crystallite (Knieke et al., 2009), but amorphous drug due to the modified molecular arrangement might theoretically reflect different behaviors of particle size reduction when it was exposed to external forces. Glibenclamide nanosuspensions was produced and systematically investigated by using freeze-dried API with various reduced crystallinities in the method of design of experiment (DoE). The optimized particle size distribution was obtained from amorphous freeze-dried powder with porous morphology after HPH process (Salazar et al., 2011). In another study, resveratrol was modified by using three different methods (spray-drying, rotary evaporation and quench-cooling) and was further nanosized by HPH. Interestingly, the smallest particle size of nanosuspension was produced from the spray-dried sample without significantly reduced crystallinity compared to the amorphous sample obtained after rotary evaporation (Liu et al., 2018). Although solid state could play a part according to the published literature, the selection of modification approach seemed also important in order to produce the optimized powder for followed top-down process.

### 2.2. Ultra-small scale for initial research stage

Ultra-small production scale means further minimized drug load requirement for production compared to normal lab scale. Reduction of drug amount, especially for WBM, is valuable for API at initial research stage when the available quantity is limited or the drug is very expensive. For the lab scale WBM, a magnetic stirrer coupled with beaker or glass bottle containing beads could be used as a substitute for simulating of milling instrument. In one study about ultra-small scale production, the milling drug weight per batch can be minimized to only 5 mg by using a 1 ml milling chamber coupled with a shaker, which could be used to select stabilizer in a time as well as material saving manner. The obtained particle sizes of nanosuspension were proven to be comparable to those produced from standard lab scale experiments (Liu et al., 2017). Similar studies were also found in some recent literature (Romero et al., 2016; Hagedorn et al., 2019).

### 2.3. Reduced crystallinity of drug nanocrystals for improved bioavailability

#### 2.3.1. Reduced crystallinity of drug nanocrystals by using bottom-up

Amorphous or reduced crystallite nanosuspension can also be achieved by using some modified bottom-up approaches. The bioavailability of this kind of pure drug nanoparticle is often improved because of both its reduced particle size and low crystallinity. Nano-amorphous drug could possess better in vitro and in vivo performances than the nanocrystals as well as micro-sized amorphous formulations (Gajera et al., 2019). By itself, top-down methods do not lead to reduced the crystallinity of API due to the existence of water (T_g_, −130 °C) (Sharma et al., 2009). Bottom-up methods are the most reported approaches to produce amorphous nanosuspension (Jog & Burgess, 2017). In order to keep the amorphous state, the preparation process must be accurately controlled to avoid recrystallization and particle growth (Lindfors et al., 2007).

For example, amorphous amphotericin B nanoparticles were achieved by using precipitation. Process parameters, such as stabilizer concentration, temperature, stirring speed and time, and drug concentration were investigated and optimized to obtain the amorphous amphotericin B nanoparticles with a particle size of 135 nm (Zu et al., 2014). Besides the classic precipitation process, recently some special designed bottom-up methods were also used to produce amorphous nanosuspensions. Nanoporous membrane extrusion (NME) method is a technology, which pumped drug in solvent phase through a nanoporous membrane into the receiver solution. A particle size of 100 nm amorphous nanosuspensions could be obtained in the receiver solution. The nanoporous membrane played an important part in particle size reduction process and also avoided the aggregation (Guo et al., 2013).

#### 2.3.2. Reduced crystallinity of drug nanocrystals by using downstream

In general, solidification processes such as spray-drying or freeze-drying would not change the solid state of drug nanosuspension. However, recent studies found that drug solid state of nanosuspension could also be modified during the downstream processes. Freeze-drying is a technology, which can be used to gently dry drug nanosuspensions, preserve the particle size and improve the physical and chemical stability at the same time. Recently, it was also reported that this technology could be also employed to reduce the crystallinity of dexamethasone and tacrolimus nanosuspensions. It was proven that the obtained saturation solubility could be affected by both particle size and the crystallinity in this study (Colombo et al., 2017).

On the other side, special attention should be paid to the approach of correctly measuring the solid state of nanosuspension. One study in our research group revealed that DSC as a commonly used solid state technology was not suitable for characterization of dried nanosuspension when the API had a solubility in coexisting stabilizer, which might be concluded an inconsistent result with PXRD or other measurements (Liu et al., 2018).

The hot-melt extrusion is another way to process the obtained nanosuspensions. For example, the poorly soluble drug, efavirenz, was first prepared by HPH to achieve drug nanosuspension, which was then mixed with polymer Soluplus^®^ using a twin-screw extruder to get nanocrystal solid dispersions. 6 months’ stability was confirmed. The increased dissolution rate was achieved using this methods resulted not only from the reduced particle size but also from the improved wettability due to the intimate polymer contact (Ye et al., 2016). In another study, hot melt extruder coupled with polymer matrix were used to dry amorphous nanosuspension obtained from bottom-up approach. The solubility improvement afterwards could be attributed to the nanosized API as well as the applied polymer matrix (Gajera et al., 2018).

### 2.4. Nanocrystals loaded in carrier (or carrier-free) for controlled drug release and targeting

#### 2.4.1. Carrier-free (stabilizer-based) nanocrystals

**Figure 3.**
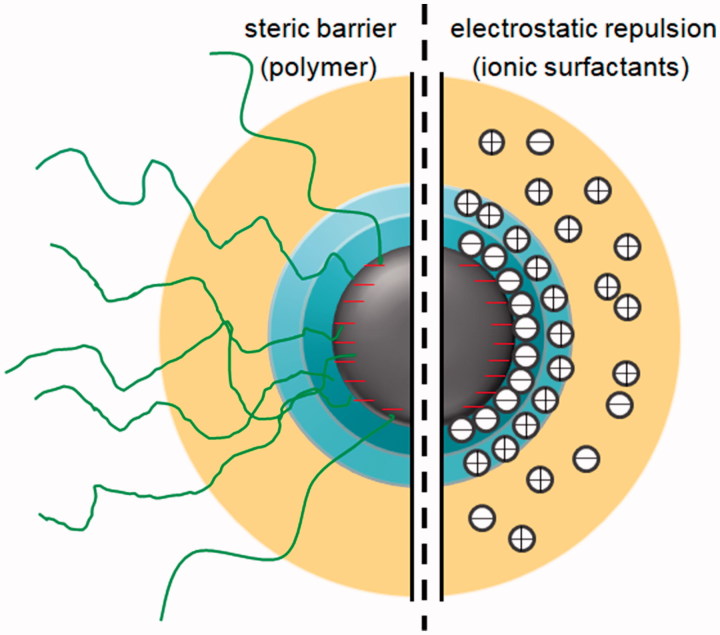
Schematic description of two different stabilizer functions in regards to steric barrier and electrostatic repulsion.

Stabilizer could not only affect the stability of obtained nanosuspension but also the efficiency of particle size reduction as well as in vivo and in vitro performances. The commonly used stabilizer could be mainly divided into two types, i.e. ionic surfactants (sodium cholate, sodium dodecyl sulfate and so on) and polymer (hydroxypropyl methylcellulose, polyvinyl alcohol and so on), which are all basically pharmaceutical excipients and can be used individually or in combination. Electrostatic repulsion or steric barrier for the two kinds of stabilize are the reason that could prevent the aggregation of nanocrystals, respectively (Yang et al., 2018). Figure 3 gives a schematic description of the two different mechanisms. In addition, the selected stabilizer could also have influence on the bioavailability and a recent study in 2019 found that combination of ionic surfactant and polymer could highly contribute to the bioavailability (Wang et al., 2019). Some stabilizers such as TPGS and poloxamers are P-gp inhibitors and could reverse the drug resistance (Tuomela et al., 2016). For example, Paclitaxel nanosuspension with TPGS significantly prevented the drug resistant of P-gp for H460 human cancer cells and was observed five times higher tumor inhibition than the paclitaxel solution (Gao et al., 2014). Tween 80 as the nanosuspension stabilizer could contribute to the transportation of curcumin to brain in the way of receptor-mediated endocytosis (Dibaei et al., 2019). In a recent study, the charges of stabilizers were proven the effect on the mucoadhesiveness and permeation of cell monolayers, which confirmed that positively charged nanosized clarithromycin was observed better mucoadhesiveness than the not charged and the negatively charged nanocrystals, and both charged nanocrystals reflected an improved drug delivery compared to the uncharged (Soisuwan et al., 2019). In another study, muco-inert Pluronic F127 as the stabilizer for budesonide nanosuspension was proven to lead to highly decreased number of inflammatory macrophages compared to the microsized budesonide, when model mouse were given enema (Date et al., 2018). In addition, molecular weights of the applied polymer could also affect the in vivo oral absorption. It was found that the fenofibrate nanocrystals stabilized by low molecular weight hypromellose (HPMC) could transport the mucin faster than the large one and therefore possessed optimized oral absorption (Ueda et al., 2019).

Novel stabilization strategies are usually developed aiming to find the substitution for the above mentioned stabilizer and possess improved stability or specific function. For example, baicalin nanocrystals were homogenized by using a surfactant free method, which applied a combination of nanocrystalline cellulose and sodium carboxymethyl starch as co-stabilizer. In vitro dissolution and in vivo bioavailability were both observed superior compared to the raw API and the redispersibility was also benefited after the spray-drying process (Xie et al., 2019). In another study, alginate was used as stabilizer for HPH, which was an anionic polysaccharide and possessed both electrostatic and steric effects. The main advantage of this stabilizer was the low application amount in nanosuspension, which was only 0.5–1% (*w*/*w*) of the API (Guan et al., 2017).

Same as the stabilizer used for top-down process, the stabilizer within the bottom-up processes can also not only affect the stability of obtained nanosuspensions but also the production efficiency and in vitro as well in vivo performance. Besides widely used pharmaceutical stabilizer such as HPMC, PVP, sodium cholate and Tween 80, some novel stabilizer, e.g. food protein (soybean protein) was attempted, which showed improved stabilization efficiency compared to traditional stabilizers. The obtained nanosuspensions could be further freeze-dried and showed good redisperseability (He et al., 2013). In another study, a biocompatible amphiphilic block copolymer poly (L-phenylalanine)-b-poly(L-aspartic acid) was used as the stabilizer for a combination of precipitation and HPH process to produce paclitaxel nanosuspension, which was then proven to significantly decrease the plasma peak concentration and prolong plasma circulating time. In addition, this stabilizer could contribute to the drug penetration of multidrug resistance cells (Cao et al., 2019).

Stabilizer as one of the most important parts of nanosuspension for both top-down and bottom-up methods (Zhou et al., 2018), it was found that more and more naturally occurring materials from plants or animals are used as the novel because of their good biocompatibility according to the recent studies. In addition, modified chemical surfactants or polymers by using organic synthesis are another large group of advanced stabilizers that could achieve improved stability or controlled release.

#### 2.4.2. Nanocrystals loaded in carrier

Drug nanosuspensions can be further converted to not only tablets (Mori et al., 2016; Anup et al., 2018), capsules (Bonhoeffer et al., 2018), creams (Lai et al., 2015), injectables (Lu et al., 2016; Yang et al., 2017; Sigfridsson et al., 2019) or other traditional formulations (Pailla et al., 2019), but it can be also loaded in carrier such as human erythrocytes (Staedtke et al., 2010) or be combined with fluorescent dyes (Zhao et al., 2011) with the development of modern pharmaceutical technologies. This review would mainly focus on recently developed novel approaches because the traditional methods have been summarized in many related literature (Van Eerdenbrugh et al., 2008; Malamatari et al., 2016).

Although drug nanocrystals is a carrier free system, it usually needs an approach or medium for the drug delivery. Drug nanocrystals can be further loaded in novel carriers, which sometimes can significantly alter their physical properties and subsequently lead to modified release (Wei et al., 2018). Many recently related publication showed that loading nanocrystals in different new carriers is an attractive research direction. Besides traditional tablet or capsule, novel carriers can also be cell or specific designed material, such as gel or film. Examples are listed as follows.

##### 2.4.2.1. Nanocrystals loaded in carriers for oral

Nanocrystals can be loaded in oral dosage such as common tablet or capsule. There are different ways to handle the obtained nanosuspensions. Normally, drug nanosuspension could be dried by using freeze-drying (Alshweiat et al., 2018) or spray-drying (Nair et al., 2019) to produce powder, which then is applied for tabletting or other oral formulation. Alternatively, nanosuspension could be also directly used as ingredient of formulation for downstream process. For example, nanosuspension could be used as wet agent to mix with other excipients such as starch for granulation and then was applied for tabletting (Liu et al., 2018). In one study, glyburide nanocrystals were loaded in lactose monohydrate and microcrystalline cellulose for tabletting, which was finally observed highly improved in vivo AUC value compared to the marketed tablets (Ali et al., 2019).

Recently, fluidized bed can also be used to dry drug nanosuspensions and simultaneously coat them onto sugar spheres. These layered cores can be further coated with functional polymers for to achieving e.g. controlled drug release properties. For example, naproxen and cinnarizine nanosuspensions were processed by a fluidized bed pellet coater. Both showed improved dissolution compared to their raw APIs. In this study, it was confirmed that drug physicochemical properties have important effect on dissolution behavior after laying processes, which could not be predicted from the information of nanosuspensions (Kayaert et al., 2011).

In addition, drug nanocrystals could be incorporated into oral carrier such as fast dissolving films. Lai et al. applied HPH to prepare quercetin nanosuspensions and then a casting method was used to prepare the film (maltodextrins as film forming material). This downstream method could effectively avoid the physical stability issue related to nanoparticlate systems. Interestingly, the nanocrystal loaded films showed much faster dissolution rate compared to the freeze-dried nanocrystals (Lai et al., 2015).

##### 2.4.2.2. Nanocrystals loaded in carriers for inhalation

Nanocrytals are homogenous small drug particles, which are suitable to be loaded in microparticle by using instrument such as spray-drying for inhalation and improve the corresponding bioavailability as well as the adhesion of the poorly soluble drug owing to the enlarged drug particle surface. The microparticle as a carrier for nanocrystals might be consisted of mannitol or other pharmaceutical excipient.

Most of the inhalation related nanocrystal products were designed for immediate functions, however, recent studies found that nanocrystals loaded in polymer such as hyaluronic acid could lead to continuous drug release and improved bioavailability after administration. The polymer or carrier used could prevent mucociliary clearance and consequently resulted in extended drug retention, where a shrinking of the dissolution rate was not essential (Liu et al., 2018). In another study, it was found that the stabilizer used for HPH could significantly affect the in vivo pharmacokinetic performance. For example, one study achieved resveratrol nanocrystals loaded in mannitol based microparticles for inhalation and positively charged surfactant chitosan contributed to the drug retention and maximum avoided the API entrance of the general circulation (Liu et al., 2019).

##### 2.4.2.3. Nanocrystals loaded in carriers for transdermal

Drug nanocrystals could be loaded in commonly used such as gel (Barkat et al., 2017; Kumar et al., 2019) to achieve transdermal administration, which could lead to improved in vitro and in vivo performances compared to the microsized API formulation or drug solution (Oktay et al., 2018). For example, silver sulfadiazine nanosuspension was produced by HPH to obtain an average particle size of 369 nm and then it was loaded in thermosensitive hydrogel. The drug release results showed obvious improvement of the loaded sample compared to the commercial product and the in vitro antibacterial experiment also was found superior for the nano-silver sulfadiazine formulation (Liu et al., 2018). Some combinations of nanocrystals with specified designed carriers have been reported.

Microneedle is an advanced and painless drug delivery technology, which has been recently combined with nanosuspension (Vora et al., 2018; Permana et al., 2019). Rapidly dissolving microneedles, which could be fully dissolved and degraded in the skin (Sullivan et al., 2008), is difficult for poorly water soluble compound to be applied due to the distribution problem. Therefore, nanosuspension as a homogenous system can used to remedy this defect. On the other side, the poor permeation ability of nanosuspension for stratum corneum could be dissolved by combined with mirconeedles. Abdelghany et al applied curcumin as model compound and the obtained nanocrystals loaded microneedles could reach 500 µm in neonatal porcine skin and dissolved within 60 min, afterwards, lead to a depth of 2300 µm, which was more notable than the simple curcumin nanosuspension (Abdelghany et al., 2019). In addition, a long term acting rilpivirine nanosuspension in microarray patches was achieved and investigated, which showed that the API plasma concentration in rats was about 10 times higher than former researches. Moreover, rilpivirine could be found in lymph nodes, which means this antiretroviral API could be transported to one important site of HIV duplication during prolonged duration (McCrudden et al., 2018).

##### 2.4.2.4. Nanocrystals loaded in cell for targeting

Human cells, unlike synthetic chemical carrier, are considered as biocompatible and have been applied for the drug carriers (Tan et al., 2015). Cells, such as red blood cells, leukocytes or stem cells have been reported to be used as carriers for nanoparticle to get required drug target (Pang et al., 2017). However, nanocrystals generally possessed larger particle size range of 100–1000 nm than other nanoparticle systems (normally smaller than 100 nm). Therefore, it is essential to produce a smaller particle size in order to combine with cell carriers. For example, amphotericin B is a drug with poor solubility, low in vivo stability and high toxicity. In order to overcome those problems, amphotericin B nanocrystals were firstly prepared by the H 96 technology (FD combined by HPH). A smaller particle size (i.e. 65 nm) was obtained than one-step HPH process. The achieved nanocrystals were then encapsulated in human erythrocytes by applying hypotonic hemolysis, where finally could effectively prevent the drug’s toxicity on the phagocytosing cells (Staedtke et al., 2010).

### 2.5. Printing nanosuspension for personalized medicines

Pharmaceutical printing technology was developed aiming personalized medicines to meet the need of patient individual dosing options, which possesses advantages such as accurate control of drug content and convenience of application. The number of publication regarding as has increased significantly recently, which indicates the rising popularity of this technology. Normally, solution (organic or water based) containing API is used for the printing process (Alomari et al., 2015). In one study, 10% (w/w) folic acid nanosuspension as printing ink could be printed on edible paper carriers via inkjet-type printing technique. This study confirmed that particle size of drug nanocrystals is small enough to be used as ink without clogging. The obtained product had advantages of both improved dissolution rate as well as the characters of personalized medicines (Pardeike et al., 2011).

### 2.6. Hybrid nanocrystals for drug tracking

Drug nanocrystals tracking in vivo is difficult because of the ongoing dissolution and limited usable method for detection. Hybrid nanocrystals are the combination of API and fluorescent dyes (Lu et al., 2017, 2019; Mohammad et al., 2019), which was invented to track nanocrystals after adiminstration. It can be achieved by using bottom-up methods. Firstly, dyes, such as fluorescein and rhodamine B were dissolved in water as antisolvent and then it was mixed by the drug solution. Hybrid drug nanocrystals possess fluorescence because of the attached dyes (Zhao et al., 2011). This technology was designed for in vivo disease imaging, such as the drug distribution during cancer treatment (Hollis et al., 2014). For example, curcumin was hybridized with fluorescent dyes from crystal lattices, which was injected intravenously. Because of the coupled fluorescent dyes, it could be shown that most API was cleared from blood very fast and accumulate predominately in liver and lung (Wang et al., 2018). In another study, an anti-HIV drug 5-Chloro-3-phenylsulfonylindole-2-carboxamide was coupled with fluorescent dyes to formulate hybrid nanocrystals for vaginal delivery and it could be imaged by confocal microscopy that nanocrystals stayed on the surface of the cell membranes at the beginning and then transported into the cytoplasm (Gong et al., 2019).

In addition, poorly water soluble dyes, such as nile red could also be directly nanosized by WBM, which could be applied to study the nanocrystal penetration and accumulation in the hair follicles with the assistance of fluorescence imaging as well as microscopy technology (Corrias et al., 2017).

## 4. Conclusion

Drug nanocrystals is simple but effective drug delivery approach for improvement of bioavailability for poorly soluble APIs. The developing trend regarding as is changing to modification of the pure drug nanocrystals by applying various novel preparation or downstream processes for specific required drug delivery approaches according to most recent studies. These modifications take advantage of the simple composition of drug nanosuspension and also endue it with improved or modified properties, such as controlled release, in vivo disease tracing or targeting. Some of them demonstrated more advantages compared to traditional pure nanocrystals. However, high cost and complicated process operation are still the main hurdles for a broader application of some of the new technologies. The development of the pharmaceutical technologies and increased various requirements from the clinical would lead to the appearance of more practical tailor-made nanocrystals.
